# Effects of Zinc Sulfate Supplementation in Treatment of Iron Defi ciency Anemia

**DOI:** 10.4274/Tjh.2012.0043

**Published:** 2013-06-05

**Authors:** Meltem Gülsan, Barış Malbora, Zekai Avcı, Nilüfer Bayraktar, İkbal Bozkaya, Namik Özbek

**Affiliations:** 1 Başkent University Faculty of Medicine, Department of Pediatrics, Ankara, Turkey; 2 Dr. Sami Ulus Maternity and Children’s Hospital, Department of Pediatric Hematology, Ankara, Turkey; 3 Başkent University Faculty of Medicine, Department of Pediatric Hematology, Ankara, Turkey; 4 Başkent University Faculty of Medicine, Department of Biochemistry, Ankara, Turkey; 5 Ankara Pediatric Hematology and Oncology Hospital, Department of Pediatric Hematology, Ankara, Turkey

**Keywords:** Iron deficiency anemia, treatment, Ferrous sulfate, Zinc sulfate

## Abstract

**Objective:** In this study, we aimed to compare the effect(s) of zinc sulphate on growth and serum iron variables when it is given with ferrous sulphate in iron deficiency anemia (IDA).

**Materials and Methods:** Patients (n=79) were randomly divided into two groups. In one group (n=40) 4 mg/kg/d ferrous sulfate was given orally. In the other group (n=39), in addition to ferrous sulfate, 5 mg/d oral zinc sulfate was given.

**Results:** Compared to the initial values statistically significant increase in mean height, weight, and head circumference has been observed in both groups after 3 months. However, there was no statistical difference between two groups concerning mean height, weight, and head circumference at the beginning (83.43±11.3 cm vs 84.62±12.77 cm; 12.36±3.08 kg vs 12.72±3.87 kg; 47.33±2.15 cm vs 47.26±2.73 cm, respectively), at the first month, (84.82±10.97 vs 85.97±12.28; 12.78±3.09 vs 13.09±3.87; 47.76±2.10 vs 47.61±2.67, respectively), and at the third month, (86.4±11.12 vs 87.69±12.13; 12.9±3.06 vs 13.35±3.81; 48.22±1.89 vs 48.07±2.45, respectively). There were no statistical differences between mean hematological parameters of the groups at the beginning, at the first month, and at the third month, either (mean hb of Group 1: 8.78±1.12 g/dL; 11.27±1.09 g/ dL; 12.05±1.00 g/dL respectively and of Group 2: 9.10±1.07 g/dL; 11.12±0.85 g/dL; 11.80±0.79 g/dL, respectively). Mean ferritin and zinc values of the groups were statistically insignificant at the beginning (Mean ferritin: 4.96±4.03 μg/dL vs 4.52±2.94 μg/dL, zinc: 88.64±15.35 ng/mL vs 86.84±17.34 ng/mL). Their increase was statistically significant at the third month (mean ferritin: 15.91±9.57 μg/dL vs 15.25±10.47 μg/dL; zinc: 88.02±15.10 ng/mL vs 95.25±16.55 ng/mL).

**Conclusion:** In our study neither positive nor negative effect of zinc administration on IDA treatment was demonstrated. Therefore, in the treatment of IDA zinc together with iron should be used at different times if there is coexistent zinc deficiency.

**Conflict of interest:**None declared.

## Abstract (tr)

**Amaç:** Bu çalışmada, demir eksikliği anemisi tedavisinde ferröz sulfat verildiğinde çinko sülfatın büyüme ve serum demir değişkenleri üzerine etkinliğini karşılaştırmayı amaçladık.

**Gereç ve Yöntemler:** Hastalar (n=79) rastgele iki gruba ayrıldı. Bir gruba (n=40) 4 mg/kg/gün’den oral yolla ferröz sülfat verildi. Diğer gruba ise (n=39) ferröz sülfata ek olarak 5 mg/gün’den çinko sülfat verildi.

**Bulgular:** Başlangıç değerleri ile karşılaştırıldığında tedavinin 3. ayında her iki grupta ortalama boy, vücut ağırlığı ve baş çevresi açısından istatistiksel olarak anlamlı bir artış tespit edildi. Ancak, iki grup arasında ortalama boy, vücut ağırlığı ve baş çevresinde tedavinin başlangıç (sırasıyla 83,43±11,3 cm ve 84,62±12,77 cm; 12,36±3,08 kg ve 12,72±3,87 kg; 47,33±2,15 cm ve 47,26±2,73 cm), 1. ay (sırasıyla 84.82±10,97 ve 85,97±12,28; 12,78±3,09 ve 13,09±3,87; 47,76±2,10 ve 47,61±2,67) ve 3. ayları (sırasıyla 86,4±11,12 ve 87,69±12,13; 12,9±3,06 ve 13,35±3,81; 48,22±1,89 ve 48,07±2,45) karşılaştırıldığında istatistiksel bir farklılık yoktu. Ortalama hematolojik parametrelerde de iki grup arasında başlangıç, 1. ay ve 3. ay değerleri karşılaştırıldığında istatistiksel fark görülmedi (sırasıyla grup 1 ortalama Hb: 8,78±1,12 g/dL; 11,27±1,09 g/dL; 12,05±1,0 g/dL, grup 2 ortalama Hb: 9,10±1,07 g/dL; 11,12±0,85 g/dL; 11,80±0,79 g/dL). Başlangıç ortalama ferritin ve çinko değerleri gruplar arasında istatistiksel olarak farklı değildi (ortalama ferritin: 4.96±4.03 μg/dL ve 4,52±2,94 μg/dL, ortalama çinko: 88,64±15,35 ng/mL ve 86,84±17,34 ng/mL). Ortalama ferritin ve çinko düzeyleri 3. ayda istatistiksel olarak anlamlı şekilde yükseldi (ortalama ferritin: 15,91±9,57 μg/dL ve 15,25±10,47 μg/dL; ortalama çinko: 88,02±15,10 ng/mL ve 95,25±16,55 ng/mL).

**Sonuç:** Çalışmamızda DEA tedavisinde çinko verilmesinin pozitif veya negatif bir etkisi gösterilemedi. Bu yüzden, DEA tedavisinde demire ek olarak çinko farklı zamanlarda, eşlik eden çinko eksikliği varsa verilmelidir.

## INTRODUCTION

Iron deficiency, the most common cause of anemia in the world and in Turkey, is estimated to affect 2 billion people, and more than half of them are anemic [1,2]. In Turkey, a prophylaxis program for iron deficiency was started in 2004 by the Ministry of Health. According to this program, all children should receive 1 mg/kg/d of ferrous sulfate (Fe-S) between 4 and 12 months of age. After this, the prevalence of iron deficiency anemia (IDA) was reported as 7.8% of children aged 12-23 months [3]. Zinc is the second most abundant trace element in the organism other than iron. Nutritional zinc deficiency is also quite common in the world and in Turkey at rates as high as 15.7% [4,5,6]. Deficiencies of other trace elements, especially zinc, are frequently associated with IDA in developing countries and in Turkey [7,8,9]. Therefore, in the treatment of patients with IDA, zinc, in addition to iron, may be considered [10,11]. Because there are some concerns about their interactions in absorption from the intestines [12,13], in the present study, we aimed to compare the effect(s) of zinc sulfate (Zn-S) on growth and serum iron variables when it is given simultaneously with Fe-S.

## MATERIALS AND METHODS

**Subjects**

A total of 103 children (aged from 6 months to 6 years) admitted to the hospital with IDA between November 2009 and March 2010 were included in the study. Inclusion criteria were hemoglobin (Hb) levels below 10 g/dL, serum ferritin levels below 10 ng/mL, and mean corpuscular volume (MCV) below the standard values determined by age and sex [14]. Patients whose serum zinc levels were <70 mg/ dL were diagnosed with zinc deficiency. Patients who had any chronic disease were excluded. Presenting symptoms; dietary patterns including breast milk, cow’s milk, red meat, and complementary food consumption; socioeconomic conditions; presence of anemia in another sibling; presence of any infection at admission or within the last month; and any drug or prophylactic iron usage were recorded using a questionnaire. At each visit, height, body weight, and head circumference in children younger than 2 years old were measured and a physical examination was performed.

Patients were randomly divided into 2 groups. In the first group (Group 1), 4 mg/kg/d ferrous sulfate (Fe-S) (Ferro-sanol B® suspension by Adeka, Turkey) was given orally twice a day. In the other group (Group 2), in addition to Fe-S, 5 mg/d oral Zn-S (Zinco® suspension by Berko, Turkey) was given at least 4 hours apart. In order to prevent possible interaction during absorption, zinc and iron were given at different times. In addition, a list of iron- and zinc- rich diet and nutritional recommendations were given to all patients’ families. Vomiting, constipation, diarrhea, and extremely disturbing abdominal pain were used as drug incompatibility criteria and the compatibility of families and children to therapy was tracked by periodic phone calls and at each visit according to their laboratory levels. Whenever the anemia improved (Hb ≥ 11 g/dL), treatment was maintained with a single dose of half of the total dosage of oral Fe-S for 1 additional month to replenish the iron stores. Zinc was given for 3 months without any change in dose in Group 2.

The study was approved by the Başkent University Research Board and Ethics Committee.

Written informed consent was obtained from the parents of the children.

**Laboratory Methods**

Before treatment, complete blood count (CBC), serum ferritin, serum zinc, serum transferrin receptor (sTfR), and C-reactive protein (CRP) levels were measured. After randomization, only CBC was measured at the first month of treatment. If the anemia did not improve (Hb < 11 g/ dL), iron treatment was carried on without reduction. After this point, CBC measurements were performed at 10-day intervals until achieving a Hb level of ≥11 g/dL. At the third month, CBC, serum ferritin, serum Zn, and CRP levels were measured in all patients.

For CBC, an automated hemocytometer (Cell Dyn 3700, Abbott, USA) was used after routine calibration. Blood smears for the confirmation of IDA diagnosis were stained with Wright stain and examined under light microscopy.

Serum ferritin levels were measured by the immunoturbidimetric method (Roche/Hitachi Modular Analytics P, Japan). Serum zinc levels were measured by the atomic absorption spectrophotometric method (Atomic Absorption Spectrophotometer AA-6701F, Shimadzu, Japan). Serum transferrin receptor levels were measured by ELISA (BioVendor Human sTfR ELISA, Czech Republic).

**Statistical Methods**

SPSS 15.0 for Windows (SPSS Inc., USA) was used for the statistical evaluation of the data. The t-test for independent samples was used for normally distributed data and the Mann-Whitney U test was used for unevenly distributed data to compare variables between the Fe-S and Fe-S+Zn-S groups. To examine the time-dependent changes during treatment, the t-test for normally distributed data and the Wilcoxon test for unevenly distributed data were used. In addition, repeated measurement of 2-way ANOVA was used to examine whether the time-dependent changes showed differences between the groups.

Statistical significance was accepted at p≤0.05.

## RESULTS

**Demographic Data**

**Figure 1:**
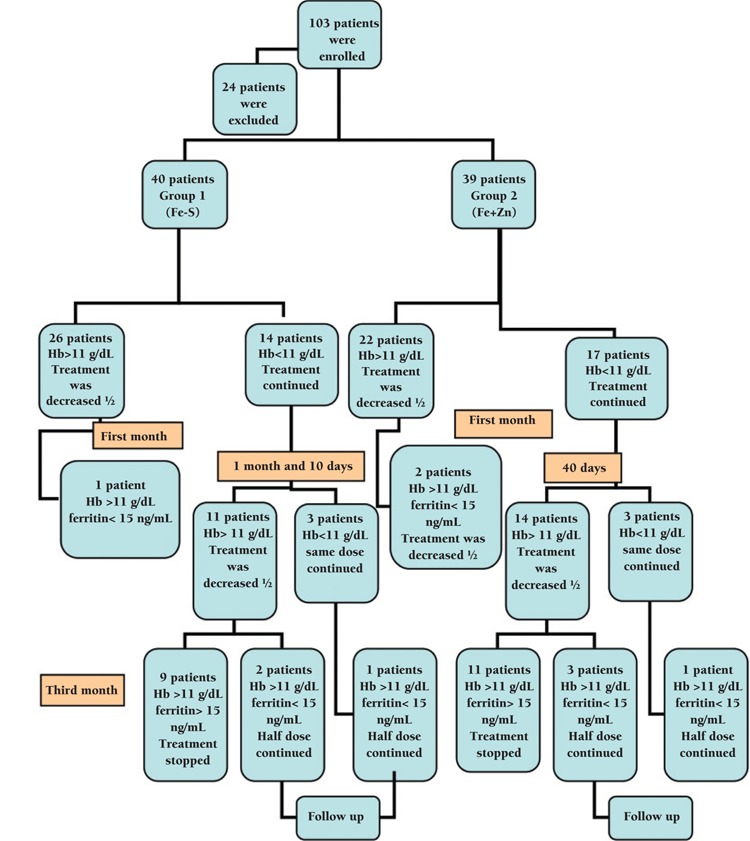
Study diagram.

Of 103 patients, 24 were excluded from study. Reasons for exclusion were serious infection of 2 patients during the study period and incompatibility with the treatment of 2 patients. Twenty patients were not accessible for follow-up. As a result, the final study population consisted of 79 patients (30 females and 49 males). Forty patients were in Group 1 and 39 patients were in Group 2. Mean ages in Group 1 and 2 were 23±16 months and 26.3±17.6 months, respectively. Groups showed an equal distribution in terms of age and sex. There were no significant differences between their socioeconomic levels (Table 1). The 2 groups also did not differ statistically in terms of family income (Table 2) and parental education level and profession (Table 1) (p=0.48, p=0.22, and p=0.91, respectively) (Figure 1).

No significant difference was found between the groups in terms of cow’s milk intake and its amount (p=0.24). In terms of duration of breastfeeding, the 2 groups did not differ significantly (p=0.6) (Table 3). Only 1 patient in Group 1 had never received breast milk, and another patient in Group 2 had been breastfed for more than 2 years. There was no difference between groups in terms of red meat consumption (p=0.65) (Table 4).

The 2 groups did not differ significantly in terms of complementary food onset time (p=0.27) (Table 5).

Two patients were exclusively breastfed (a 1.5-year-old child in Group 1 and an 8-month-old baby in Group 2). Four children had started to consume complementary foods after 1 year of age (3 children between 1 and 2 years of age, and 1 child after 2 years of age).

In our study group, 47 of 79 patients (59.5%) had not received iron prophylaxis. Seven patients older than 1 year of age had received regular prophylaxis and 11 patients had received irregular prophylaxis up to age 1. Thirteen patients had received prophylaxis regularly for 1-2 months. Only a 10-month-old baby was still receiving iron prophylaxis at the time of diagnosis. There was no difference for receiving iron prophylaxis between the 2 groups (p=0.63).

**Anthropometric Measurements**

Statistically significant increase in mean height, weight, and head circumference was observed in both groups after 3 months of treatment compared to the initial values (p<0.0001 for all 3 parameters) (Table 6). However, there were no differences between the groups concerning mean height, weight, and head circumference at the first month and at the third month of therapy (p>0.05 for all parameters). Seven patients who had zinc deficiency (4 from Group 1 and 3 from Group 2) showed comparable growth patterns with zinc-sufficient patients.

There were no children with malnutrition in the study.

**Hematological Data**

The mean sTfR levels of patients at the time of diagnosis were 5.66±3.19 μg/mL in Group 1 and 4.598±2.64 μg/mL in Group 2. Serum transferrin receptor/log ferritin ratios of patients were 3.3 in Group 1 and 2.73 in Group 2 (p>0.05). These parameters confirmed diagnosis of IDA in all patients.

When compared with initial values, mean Hb, hematocrit (Hct), MCV, mean corpuscular Hb (MCH), and mean corpuscular Hb concentration (MCHC) values showed statistically significant increases at the end of the first and third months. Mean red blood cell count (RBC) showed a significant increase at the end of 3 months compared to the pre-treatment levels; however, there was an insignificant decrease in this value between 1 and 3 months. Red blood cell distribution width (RDW) showed a statistically significant increase between 0 and 1 months and a significant decrease between 1 and 3 months. Platelet counts gradually decreased, and this reduction was statistically significant at the end of 3 months (Table 7).

Serum ferritin and zinc levels of the groups did not differ statistically at the diagnosis (p=0.6 and p=0.62, respectively). Initially 4 patients in Group 1 and 3 patients in Group 2 had zinc deficiency.

After 3 months of treatment, a mild zinc deficiency continued in only 1 patient from Group 1 (serum zinc level = 67 μg/dL). At the third month of treatment, the mean serum ferritin values increased to 15.91±9.57 ng/mL (range: 3.5-41.1 ng/mL) in Group 1 and to 15.25±10.47 ng/mL (range: 1.7-42.5 ng/mL) in Group 2. These increases were statistically significant (p<0.0001 for both).

**Figure 2:**
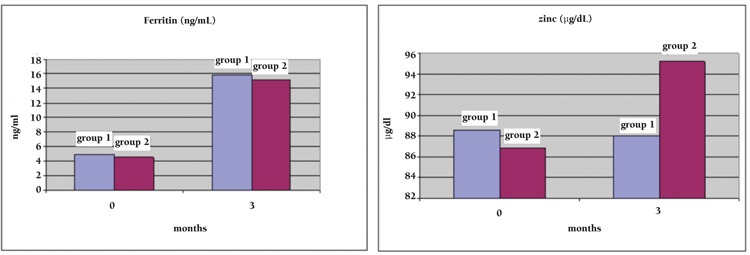
Distribution of the serum ferritin and zinc levels of the groups by months (initial and final levels).

However, there was no significant difference between groups in terms of mean serum ferritin levels at the third month (p=0.77). At the end of the third month, the mean zinc level did not change in Group 1 compared to the values at diagnosis. However, there was a significant increase concerning mean serum zinc levels in Group 2 compared to the values at diagnosis (from 88.02 μg/dL at diagnosis to 95.25±16.55 at the third month, p=0.012). A significant difference concerning zinc levels was obtained between groups at the third month (p=0.046) (Figure 2).

At the end of the first month, Hb levels of ≥11 g/dL were achieved and treatment was decreased to half of the therapeutic dose in 48 patients [26 patients (65%) in Group 1 and 22 (56.4%) patients in Group 2]. Target Hb levels were achieved at 1 month and 10 days in 25 patients [11 patients (27.5%) in Group 1 and 14 patients in Group 2 (35.9%)], and at 2 months in 6 patients (3 patients in each group).

At the end of 3 months of treatment, 4 (10%) patients in Group 1 and 6 (15%) patients in Group 2 achieved Hb levels of ≥11 g/dL; however, their ferritin levels were <10 ng/mL. These 10 patients continued two mg/kg/d Fe-S therapy after 3 months until ferritin levels reached 20 ng/mL. This period was not longer than 1 month in any patient. There were no significant differences between Groups 1 and 2 concerning time period to achieve Hb levels of ≥11 g/dL and ferritin levels of >10 ng/mL (p=0.71).

## DISCUSSION

Since iron and zinc deficiencies are frequent problems in the developing world, association of these 2 deficiencies is expected to be high [15,16,17,18]. For this reason, zinc status has been a subject of interest in children with IDA. Drugs that include zinc together with iron have been debated in terms of treatment efficacy. Therefore, there are several studies using zinc in addition to iron in prophylaxis or treatment of IDA.

A recent study from Indonesia compared prophylactic treatment regimens of 10 mg/d of iron, 10 mg/d of zinc, or both in babies starting at 4 months of age and continuing for 6 months [19]. In that study, the percentage of babies who had anemia (Hb<11 g/dL) was higher in infants taking iron and zinc compared to babies taking only iron (46% vs. 28%; p<0.05). As a result, the authors suggested that supplementation of zinc together with iron reduced its bioavailability. Another study found that only supplemental zinc during 12 weeks in adolescent athletes reduced plasma iron levels [20]. In addition, experimental studies showed that high amounts of zinc reduced iron absorption [12,13]. However, if the Fe:Zn ratio was 2:1, there was no change in the level of iron in another study [21]. In order to reduce the possible interaction of iron and zinc based on these findings, we gave iron and zinc at different times in the present study. Because there were no differences between the hematological data of our 2 groups during and after treatment, it is seen that the iron and zinc absorptions were not affected by each other if iron and zinc were given at different times.

Comparison of iron and zinc supplementation alone or together, in terms of treatment efficacy and post-treatment zinc levels, came into question with the results of some studies. Zinc deficiency was found to be very high (82.8%) in children aged 1-12 years with the diagnosis of IDA in a study from Turkey [8]. However, another Turkish study disclosed that figure to be 28.2% [9]. In our small sample, we found 7 (8.86%) patients who had zinc deficiency together with IDA. Serum zinc levels of 3 patients who had zinc deficiency in Group 1 reached normal levels and 1 patient showed a subnormal result (67 mg/dL) at the end of 3 months of Fe-S supplementation alone. Similarly, a recent study from Turkey disclosed that zinc levels of children treated for IDA increased at the third month in patients treated with Fe-S, whereas this increase was achieved at the sixth month in a group of patients treated with ferric iron [22]. This study, together with ours, supports the idea that zinc deficiency in patients with IDA could be managed by giving only ferrous sulfate. Because the iron deficiency disturbs the mucosal structure and villi, it may cause zinc deficiency due to malabsorption [23]. After mucosal regeneration, zinc absorption also improves. This could be also due to the common dietary sources of zinc and iron or the role of zinc in erythropoiesis [18]. In addition, increased appetite of children and attention of the families to these children’s diet may contribute to increased levels of zinc during the treatment of IDA.

Schultink et al. [24] compared iron sulfate and a drug containing a combination of iron sulfate and zinc in anemic (Hb<11 g/dL) children for 8 weeks. They found no statistically significant reduction in the levels of serum zinc levels after the treatment in patients who were given only iron. Interaction effects between iron and serum zinc in clinical supplementation trials were recently reviewed [25], and in 9 of 10 reviewed trials of iron-only supplementation in young children, there was no effect of iron supplementation on serum zinc. In 4 reviewed trials, the addition of iron to zinc supplements had no adverse effect on serum zinc. In our study, zinc and iron given at different times might not have influenced each other and the increases in the serum ferritin and zinc levels at the end of treatment were satisfactory.

In the present study, mean values of height, weight, and head circumference of both groups showed a statistically significant increase after treatment, but there was no significant difference between the groups. This indicates that administration of zinc together with iron does not have an additional effect on the growth of children. Lind et al. [26] found that prophylactic doses of zinc given alone increased weight for age Z-score and knee-heel length while the iron given alone increased only knee-heel distance. They observed that the effect of combined iron and zinc on growth and development was not different from the effect when given individually. In another study from Mexico, prophylactic iron, zinc, or iron plus zinc was given to children between 18 and 36 months of age for 12 months. It was seen that the growth of all 3 groups was not different from that of the children receiving a placebo [27]. In a study by Alarcon et al. [28], no difference was found between groups in terms of growth after treatment, either. The results of our study are similar to the results of these studies. Because iron and zinc were given prophylactically in most of these studies and these children did not have iron or zinc deficiency, the treatments may not have affected the growth. Our study was made up of a group with iron deficiency, but we still could not reveal any difference between the groups in terms of growth.

In Turkey, blood counts of approximately one-fourth of children admitted to a study had been measured before the beginning of the iron prophylaxis program by the Ministry of Health and two-thirds of these were anemic [3]; the program was thus started in 2004 and IDA incidence was reduced, but in our study, adherence to prophylaxis was still not good. Most of the patients had not received any prophylaxis and only a small percentage had received it regularly. Increasing the applicability and educating parents concerning the importance of iron prophylaxis would reduce the incidence of anemia in this age group.

In conclusion, the use of iron or of iron together with zinc in the treatment of IDA showed similar effects. Because these cations were given at different times in the present study, the probable interaction at the level of absorption was prohibited. Due to the high probability of multiple mineral deficiencies in developing countries, including Turkey, additional zinc supplementation to patients with IDA may be considered useful. However, in our study neither a positive nor a negative effect of zinc administration on IDA treatment was demonstrated. Therefore, in the treatment of IDA, zinc together with iron should be used at different times if there is a coexistent zinc deficiency.

**Conflict of Interest Statement**

The authors of this paper have no conflicts of interest, including specific financial interests, relationships, and/ or affiliations relevant to the subject matter or materials included. Funding Source: This study was supported by the Başkent University Research Fund (Project Number KA09/181).

## Figures and Tables

**Table 1:**
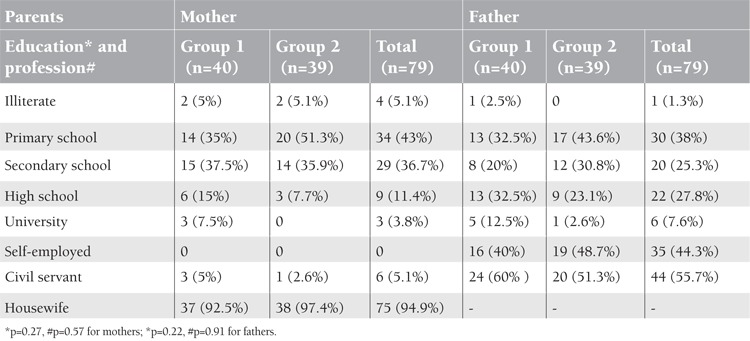
Education levels and professions of the mothers and fathers of the groups.

**Table 2:**
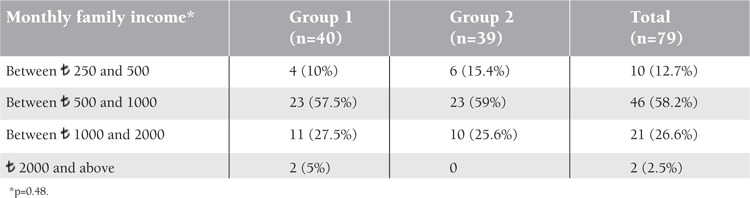
Distribution of the family income of the groups (Turkish lira, ₺ ). Subsistence wage was ₺ 521.89 and poverty line for a family of 4 people was ₺ 896 in Turkey at the study period according to the Turkish Statistical Institute (1 USD = ₺ 1.815).

**Table 3:**
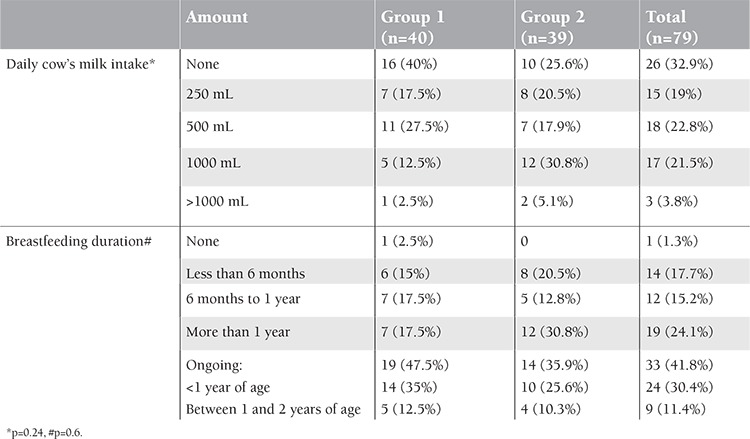
Distribution of cow’s milk intake amount and breastfeeding duration in the groups.

**Table 4:**
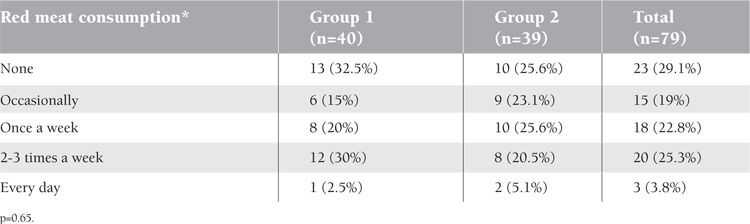
Distribution of red meat consumption of the groups.

**Table 5:**
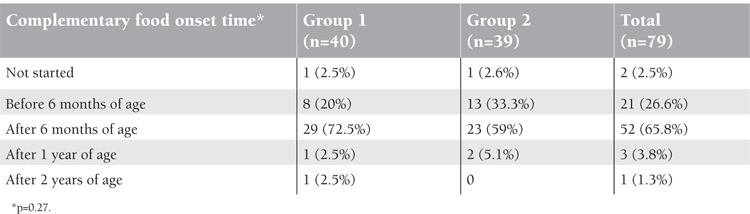
Distribution of complementary food onset time of the groups.

**Table 6:**
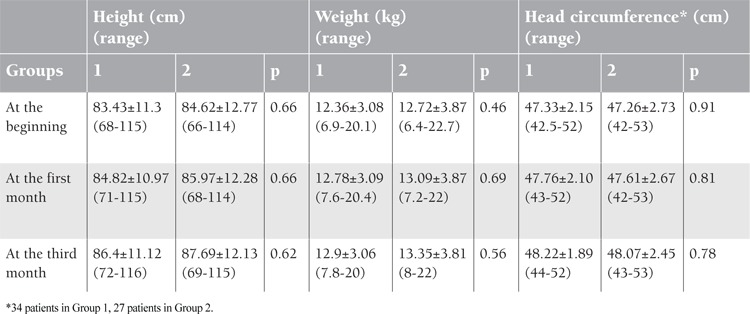
Comparison of the mean height, weight, and head circumference values of the groups at the beginning and at the first and third months.

**Table 7:**
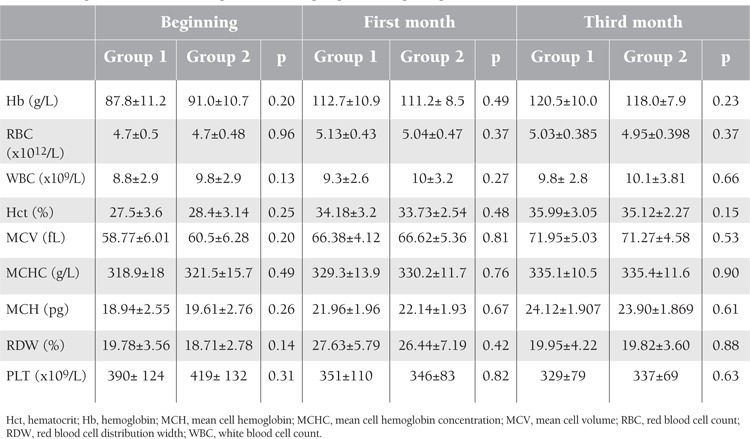
Comparison of the hematological data of the groups at the beginning and at the first and third months.
